# Supplementary material for: Predator in proximity: how does a large carnivore respond to anthropogenic pressures at fine-scales? Implications for interface area management
Source: PeerJ. 2024 Jul 10;12:e17693. doi: 10.7717/peerj.17693 (PMC11246029; doi:10.7717/peerj.17693)
Supplement: Supplemental Information 1 [file peerj-12-17693-s001.docx]

| **Distance class** | **No. of camera traps** | **No. of independent tiger captures** |
| --- | --- | --- |
| 0-1 km | 50 | 87 |
| 1-2 km | 69 | 279 |
| 2-3 km | 55 | 362 |
| 3-4 km | 17 | 150 |
| **Total** | 191 | 878 |
