# Supplementary material for: Predator in proximity: how does a large carnivore respond to anthropogenic pressures at fine-scales? Implications for interface area management
Source: PeerJ. 2024 Jul 10;12:e17693. doi: 10.7717/peerj.17693 (PMC11246029; doi:10.7717/peerj.17693)
Supplement: Supplemental Information 2 [file peerj-12-17693-s002.docx]

| **S. no.** | **Species/Group** | **No. of captures** |
| --- | --- | --- |
| 1 | Asiatic Wildcat | 327 |
| 2 | Birds | 2865 |
| 3 | Common Palm Civet | 415 |
| 4 | Dog | 661 |
| 5 | Four-Horned Antelope | 750 |
| 6 | Golden Jackal | 1903 |
| 7 | Gray Langur | 1089 |
| 8 | Grey Wolf | 73 |
| 9 | Honey Badger | 28 |
| 10 | Humans | 10436 |
| 11 | Indian Crested Porcupine | 1100 |
| 12 | Indian Fox | 291 |
| 13 | Indian Gazelle | 151 |
| 14 | Indian Hare | 4052 |
| 15 | Indian Pangolin | 12 |
| 16 | Jungle Cat | 541 |
| 17 | Leopard | 761 |
| 18 | Livestock | 3690 |
| 19 | Madras Treeshrew | 5 |
| 20 | Mongoose | 517 |
| 21 | Nilgai | 2768 |
| 22 | Rodents | 74 |
| 23 | Rhesus Macaque | 2 |
| 24 | Rusty-spotted Cat | 37 |
| 25 | Sambar | 5725 |
| 26 | Sloth Bear | 266 |
| 27 | Small Indian Civet | 967 |
| 28 | Spotted Deer | 9855 |
| 29 | Squirrel | 40 |
| 30 | Striped Hyaena | 835 |
| 31 | Tiger | 878 |
| 32 | Wild Pig | 2370 |
| **Total** | | **53484** |
