# Supplementary material for: Predator in proximity: how does a large carnivore respond to anthropogenic pressures at fine-scales? Implications for interface area management
Source: PeerJ. 2024 Jul 10;12:e17693. doi: 10.7717/peerj.17693 (PMC11246029; doi:10.7717/peerj.17693)
Supplement: Supplemental Information 3 — The models are arranged in increasing order of ΔAICc from left to right. [file peerj-12-17693-s003.docx]

| **Models →** | | | **Best-fit** | | **Models within 2ΔAICc** | | | | | |
| --- | --- | --- | --- | --- | --- | --- | --- | --- | --- | --- |
| **95% CI limits** → | | | **2.50%** | **97.50%** | **2.50%** | **97.50%** | **2.50%** | **97.50%** | **2.50%** | **97.50%** |
| **Ψ** | **Intercept** | | 0.898294 | 1.780996 | 0.888146 | 1.746207 | 0.899573 | 1.776611 | 0.902003 | 1.933551 |
|  | **Distance to villages** | | 0.220332 | 1.033629 | 0.224001 | 1.028331 | 0.216845 | 1.037430 | 0.260025 | 1.102975 |
|  | **RAI** | **Sambar** | -0.003332 | 1.434309 | 0.005460 | 1.389213 | -0.147104 | 1.368123 | 0.010919 | 1.587421 |
|  |  | **Humans** | - | - | - | - | - | - | -0.511546 | 1.528967 |
|  | **NDVI** | | - | - | - | - | -0.317348 | 0.628827 | - | - |
| **ρ** | **Intercept** | | -2.778553 | -2.582613 | -2.743178 | -2.561338 | -2.779365 | -2.583076 | -2.785685 | -2.586466 |
|  | **Effort** | | -0.016059 | 0.167164 | - | - | -0.015872 | 0.167383 | -0.012460 | 0.173249 |
|  | **Distance to** | **Villages** | 0.292010 | 0.463609 | 0.298953 | 0.468635 | 0.292304 | 0.464269 | 0.295828 | 0.472487 |
|  |  | **SH** | -0.343668 | -0.166171 | -0.317528 | -0.149103 | -0.343429 | -0.165942 | -0.345115 | -0.167751 |
|  | **NDVI** | | 0.025319 | 0.162688 | 0.026564 | 0.164127 | 0.024332 | 0.162107 | 0.024744 | 0.164077 |
|  | **RAI Humans** | | - | - | - | - | - | - | -0.082703 | 0.098033 |
|  | **Location** | | -0.506001 | -0.093890 | -0.537948 | -0.134372 | -0.503778 | -0.092037 | -0.508356 | -0.097927 |
