# Supplementary material for: Predator in proximity: how does a large carnivore respond to anthropogenic pressures at fine-scales? Implications for interface area management
Source: PeerJ. 2024 Jul 10;12:e17693. doi: 10.7717/peerj.17693 (PMC11246029; doi:10.7717/peerj.17693)
Supplement: Supplemental Information 4 — Each set of samples were first checked for uniformity and if they represented von Mises distribution using Watson’s Goodness-of-Fit test. If the sample fit the distribution, their bias-corrected concentration parameters (K) were checked. If K > 1, a parametric test could be used to detect the difference in the mean. None of the sample set fit the criteria, and hence, a (Fisher’s) non-parametric test for the median shift in tiger activity was used instead of a parametric test. See Pewsey et al. (2013) for details of test assumptions and execution. [file peerj-12-17693-s004.docx]

|  | **von Mises distribution** | | **Circular uniform distribution** | |
| --- | --- | --- | --- | --- |
| Time-period of tiger activity | Tiger activity (Dec-Feb) | Tiger activity (March-April) | Tiger activity (Dec-Feb) | Tiger activity (March-April) |
| α | 0.05 | 0.05 | 0.05 | 0.05 |
| Test Statistic | 0.7615 | 0.2341 | 3.527 | 3.6843 |
| Critical value | 0.066 | 0.079 | 0.187 | 0.187 |
| Null hypothesis | Rejected | Rejected | Rejected | Rejected |
