# Supplementary material for: Predator in proximity: how does a large carnivore respond to anthropogenic pressures at fine-scales? Implications for interface area management
Source: PeerJ. 2024 Jul 10;12:e17693. doi: 10.7717/peerj.17693 (PMC11246029; doi:10.7717/peerj.17693)
Supplement: Supplemental Information 5 — Parameter estimates (and their standard deviations generated using 10,000 Markov chain Monte Carlo iterations) are given in each cell when they were present in the model. [file peerj-12-17693-s005.docx]

| **Models** | **Kappa** | **Intercept** | **Distance to** | | **RAI** | | **WAIC** | **ΔWAIC** |
| --- | --- | --- | --- | --- | --- | --- | --- | --- |
|  |  |  | **Villages** | **SH** | **Humans** | **Livestock** |  |  |
| Best | 0.810 (0.061) | 3.030 (0.062) | -0.114 (0.032) | - | - | - | 2854.91 | 0.00 |
|  | 0.842 (0.062) | 3.029 (0.062) | -0.117 (0.036) | 0.009 (0.036) | - | - | 2857.38 | 2.47 |
|  | 0.830 (0.061) | 3.035 (0.063) | -0.117 (0.038) | 0.010 (0.036) | 0.012 (0.060) | - | 2860.91 | 6.00 |
| Null | 0.796 (0.061) | 3.020 (0.062) | - | | | | 2865.84 | 10.93 |
| Global | 0.810 (0.061) | 3.044 (0.540) | -0.117 (0.038) | 0.011 (0.036) | 0.055 (0.113) | -0.019 (0.049) | 2864.81 | 9.90 |
