# Supplementary material for: Predator in proximity: how does a large carnivore respond to anthropogenic pressures at fine-scales? Implications for interface area management
Source: PeerJ. 2024 Jul 10;12:e17693. doi: 10.7717/peerj.17693 (PMC11246029; doi:10.7717/peerj.17693)
Supplement: Supplemental Information 6 [file peerj-12-17693-s006.docx]

| **Tiger** | **Diel period** | **Median distance (metres)** | **MAD** | **Mean distance (metres)** | **SD** |
| --- | --- | --- | --- | --- | --- |
| P234-31 (M) | Dawn | 698.90 | 559.47 | 848.06 | 674.25 |
|  | Day | 703.52 | 565.23 | 870.98 | 662.63 |
|  | Dusk | 681.15 | 673.46 | 870.86 | 713.88 |
|  | Night | 760.17 | 551.19 | 929.10 | 686.40 |
| P213-63 (F) | Dawn | 1679.18 | 1010.43 | 1615.66 | 765.25 |
|  | Day | 1613.36 | 1009.33 | 1525.72 | 833.64 |
|  | Dusk | 1069.41 | 1147.84 | 1079.56 | 853.40 |
|  | Night | 1296.53 | 1113.23 | 1291.06 | 833.24 |
