# Supplementary material for: Predator in proximity: how does a large carnivore respond to anthropogenic pressures at fine-scales? Implications for interface area management
Source: PeerJ. 2024 Jul 10;12:e17693. doi: 10.7717/peerj.17693 (PMC11246029; doi:10.7717/peerj.17693)
Supplement: Supplemental Information 7 [file peerj-12-17693-s007.docx]

| **Tiger** | **Diel period** | **Median displacement (m/hr)** | **MAD** |
| --- | --- | --- | --- |
| P234-31 (M) | Dawn | 9.00 | 8.20 |
|  | Day | 21.29 | 25.35 |
|  | Dusk | 11.19 | 12.61 |
|  | Night | 12.55 | 14.11 |
| P213-63 (F) | Dawn | 10.20 | 10.33 |
|  | Day | 18.00 | 23.40 |
|  | Dusk | 20.95 | 29.43 |
|  | Night | 186.01 | 272.18 |
