# Supplementary material for: Predator in proximity: how does a large carnivore respond to anthropogenic pressures at fine-scales? Implications for interface area management
Source: PeerJ. 2024 Jul 10;12:e17693. doi: 10.7717/peerj.17693 (PMC11246029; doi:10.7717/peerj.17693)
Supplement: Supplemental Information 8 — The models are arranged in increasing order of ΔAICc from left to right. [file peerj-12-17693-s008.docx]

|  | | **Best-fit** | | **Models within 2ΔAICc** | | | | | | | | | | | |
| --- | --- | --- | --- | --- | --- | --- | --- | --- | --- | --- | --- | --- | --- | --- | --- |
| **95% CI limits** | | **2.50%** | **97.50%** | **2.50%** | **97.50%** | **2.50%** | **97.50%** | **2.50%** | **97.50%** | **2.50%** | **97.50%** | **2.50%** | **97.50%** | **2.50%** | **97.50%** |
| **Intercept** | | -3.3969 | -1.8207 | -2.6154 | -1.5060 | -2.7116 | -1.5588 | -3.1234 | -1.6851 | -2.6589 | -1.5252 | -3.4214 | -1.8366 | -3.4766 | -1.8541 |
| **Distance to** | **Villages** | -1.9678 | -0.4823 | -1.3137 | -0.3646 | -1.4411 | -0.4172 | -1.6318 | -0.3087 | -1.4802 | -0.4322 | -2.0104 | -0.5023 | -1.9885 | -0.4768 |
|  | **Villages^2** | 0.0040 | 0.9198 | - | - | - | - | -0.0880 | 0.7958 | - | - | 0.0160 | 0.9351 | -0.0094 | 0.9284 |
|  | **SH** | -1.5829 | -0.3511 | -1.5619 | -0.3690 | -1.5869 | -0.3844 | -1.6514 | -0.4353 | -1.5678 | -0.3555 | -1.6047 | -0.3766 | -1.5948 | -0.3432 |
|  | **Water** | - | - | - | - | -0.8991 | 0.0521 | - | - | - | - | - | - | -0.8316 | 0.1263 |
| **RAI** | **Humans** | - | - | - | - | - | - | - | - | - | - | - | - | -0.5483 | 0.1985 |
|  | **Livestock** | - | - | - | - | -0.7234 | 0.1456 | - | - | - | - | -0.6737 | 0.2327 | - | - |
|  | **Wildprey** | -1.8486 | 0.0646 | - | - | - | - | - | - | - | - | -1.8924 | 0.0789 | -1.8717 | 0.0329 |
|  | **Tiger** | - | - | - | - | - | - | - | - | -0.2093 | 0.7040 | - | - | - | - |
| **psi Tiger** | | 0.0073 | 1.6110 | - | - | - | - | -0.2414 | 0.9710 | - | - | -0.0614 | 1.5754 | 0.0113 | 1.6301 |
