# Supplementary figures and images for: Predator in proximity: how does a large carnivore respond to anthropogenic pressures at fine-scales? Implications for interface area management
Source: PeerJ. 2024 Jul 10;12:e17693. doi: 10.7717/peerj.17693 (PMC11246029; doi:10.7717/peerj.17693)

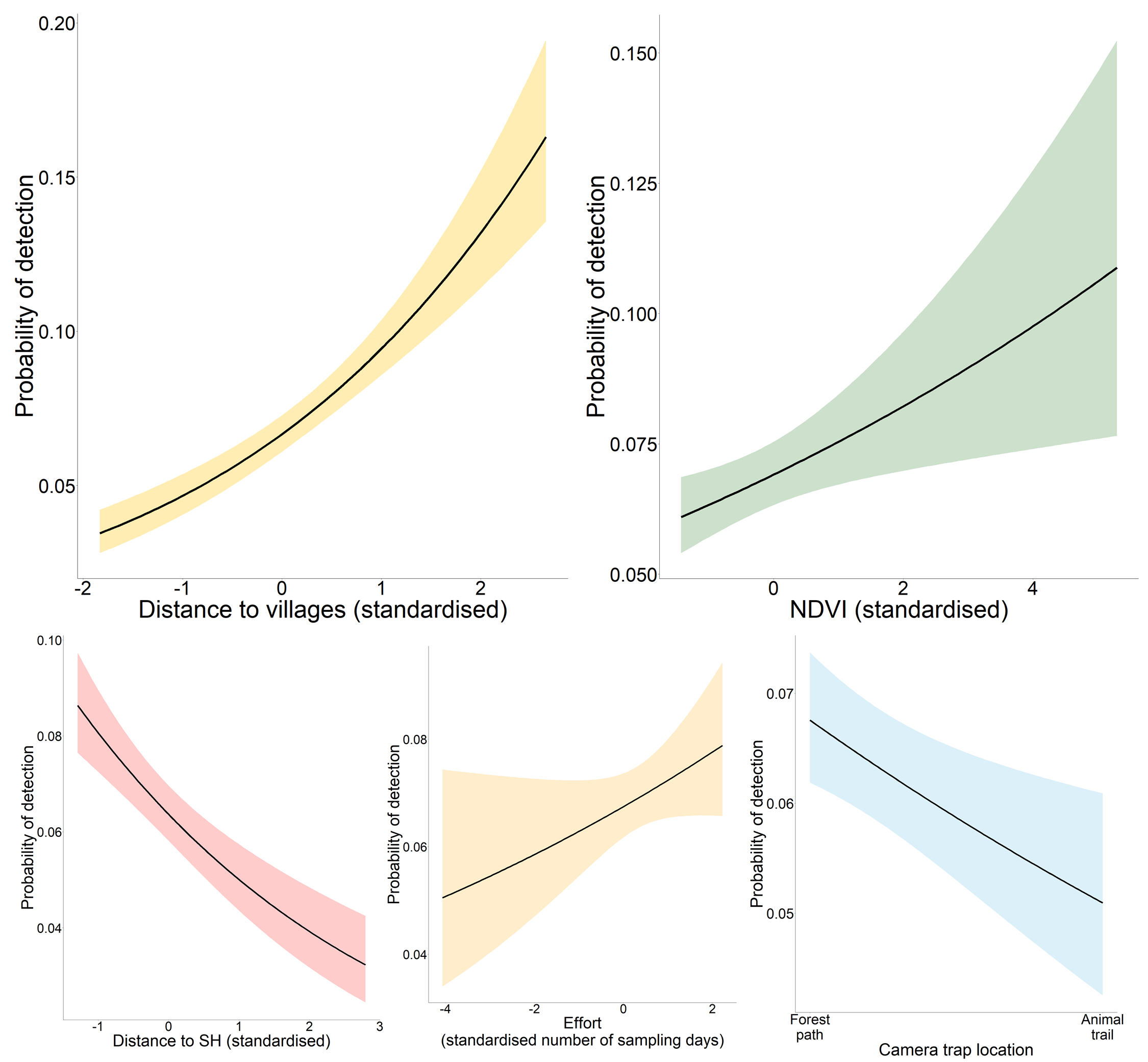

Supplement: Supplemental Information 9 — The shaded region represents the 95% confidence interval of the response curve. [file peerj-12-17693-s009.png]

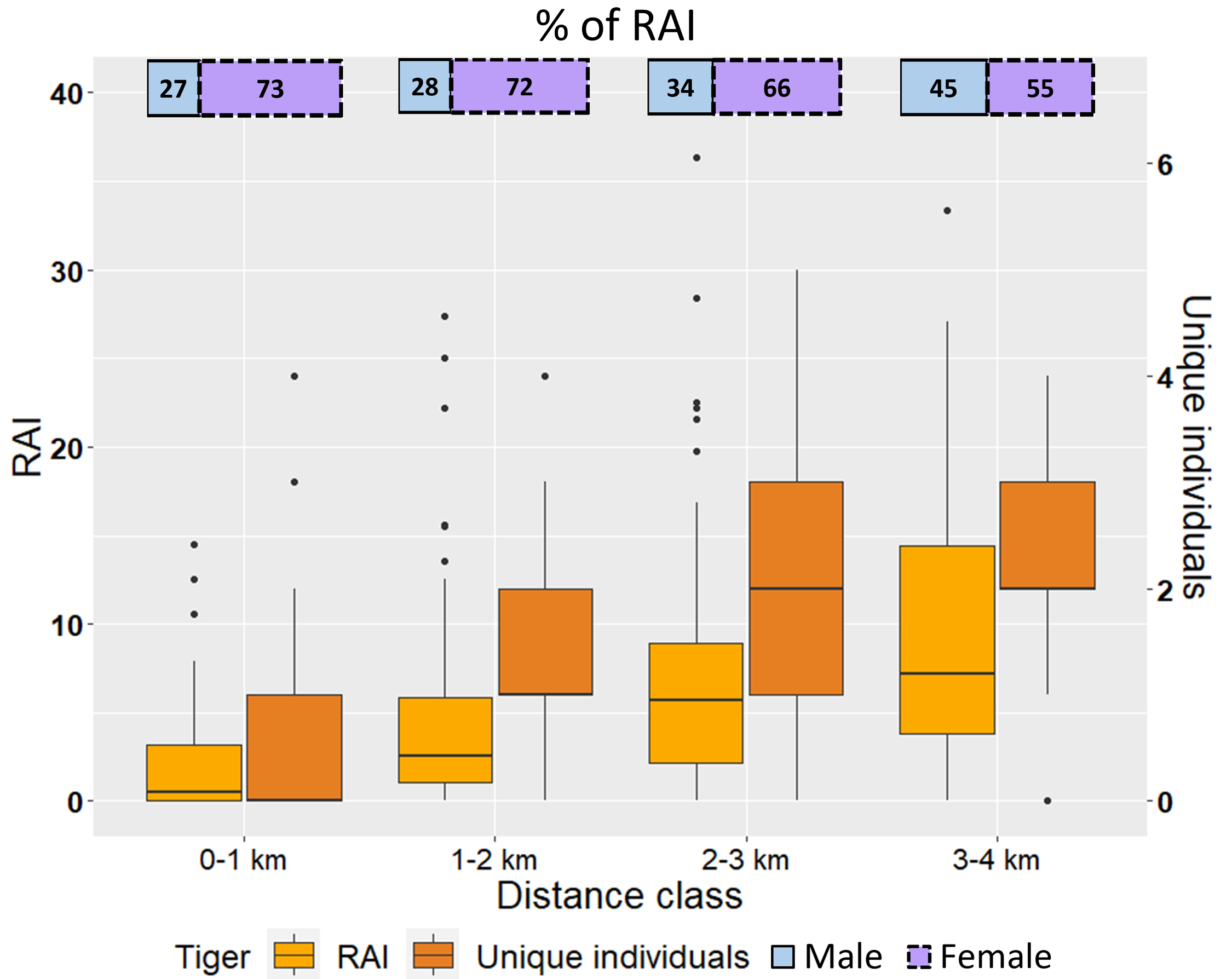

Supplement: Supplemental Information 10 [file peerj-12-17693-s010.png]

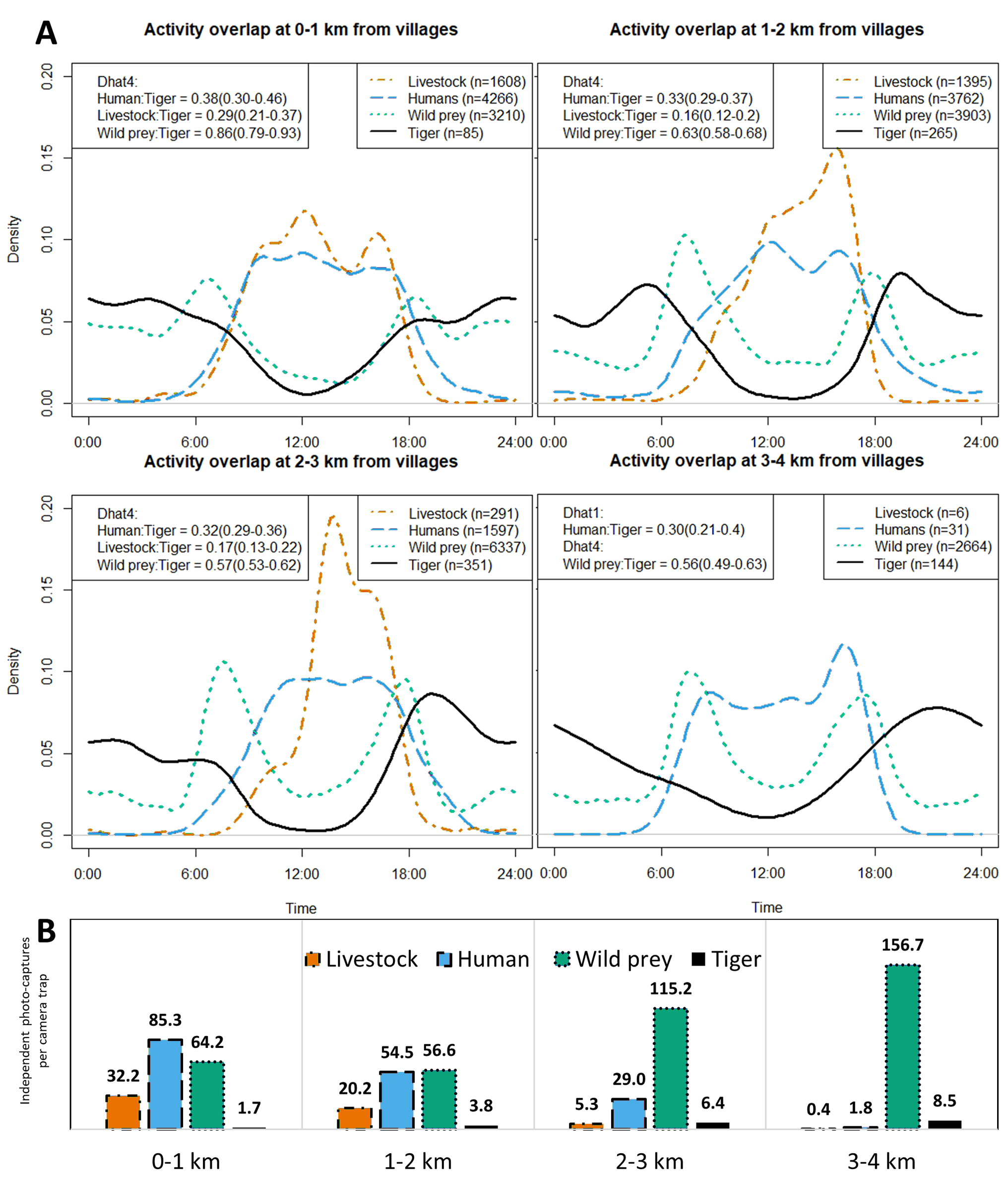

Supplement: Supplemental Information 11 — (A) (top) Activity overlap plots and (B) (bottom) bar chart of average independent photo-captures per camera trap of each species. The 95% confidence intervals of activity overlap estimate (indicated in parenthesis) are adjusted for bootstrap bias and were calculated with 10,000 resamples. Plotting and analysis of temporal overlap between tiger & livestock were avoided for the 3-4 km distance class due to very low livestock captures (n = 6). [file peerj-12-17693-s011.png]

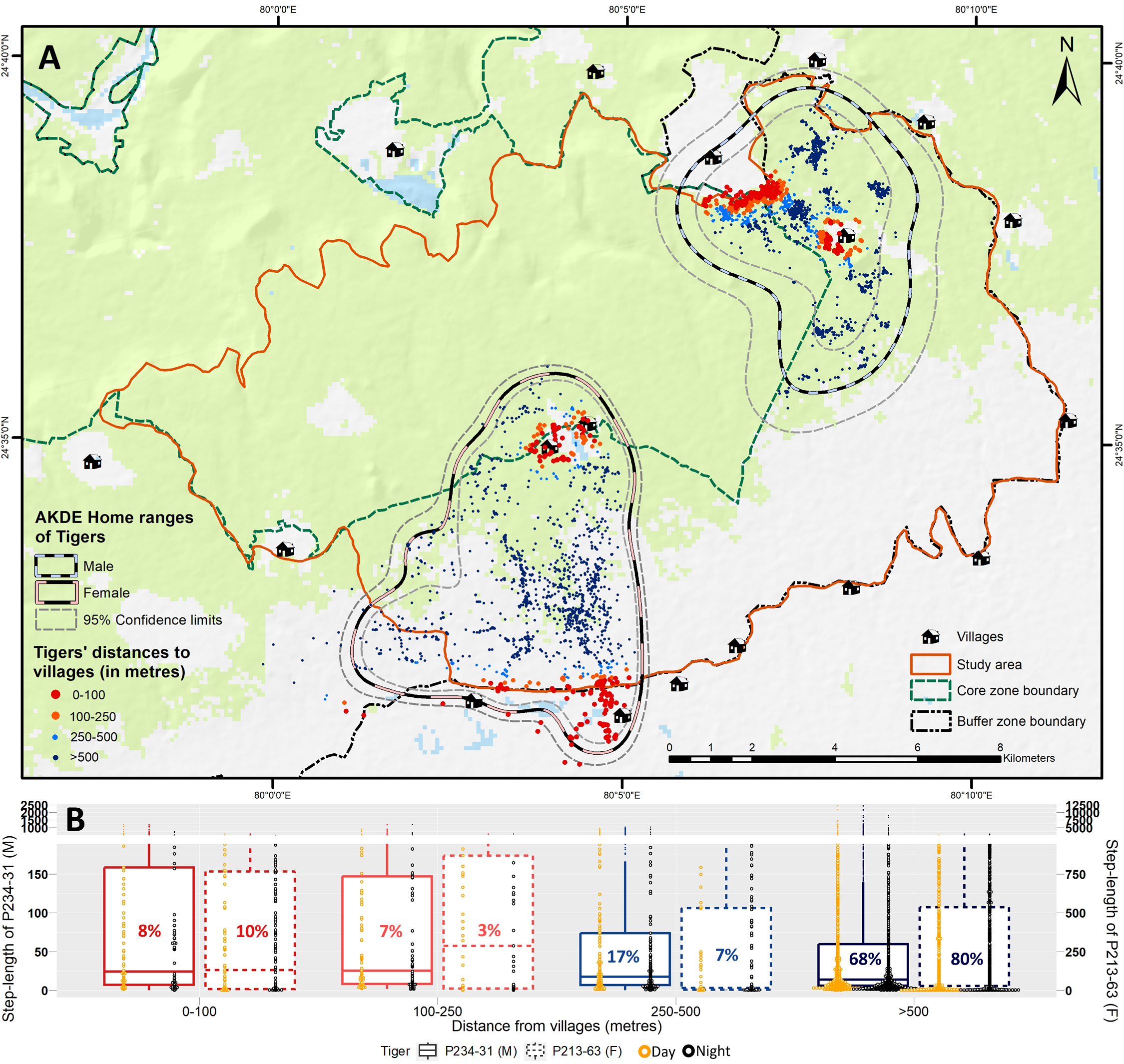

Supplement: Supplemental Information 12 — (A) (top) GPS locations, AKDE home ranges and (B) (bottom) box-whisker plots of displacement (in step-lengths: metre/hour) of the tigers at increasing distance classes from villages. Axis break has been used on the vertical axis to deal with the large number of outliers for clear visualisation and representation of the trends. The percentage values given in the boxplots are the proportion of GPS locations of the collared tigers, and the dot plots within the boxplots represent their distribution in day and night in the respective distance classes. Base layer credits: CartoDEM; FROM-GLC10. [file peerj-12-17693-s012.png]

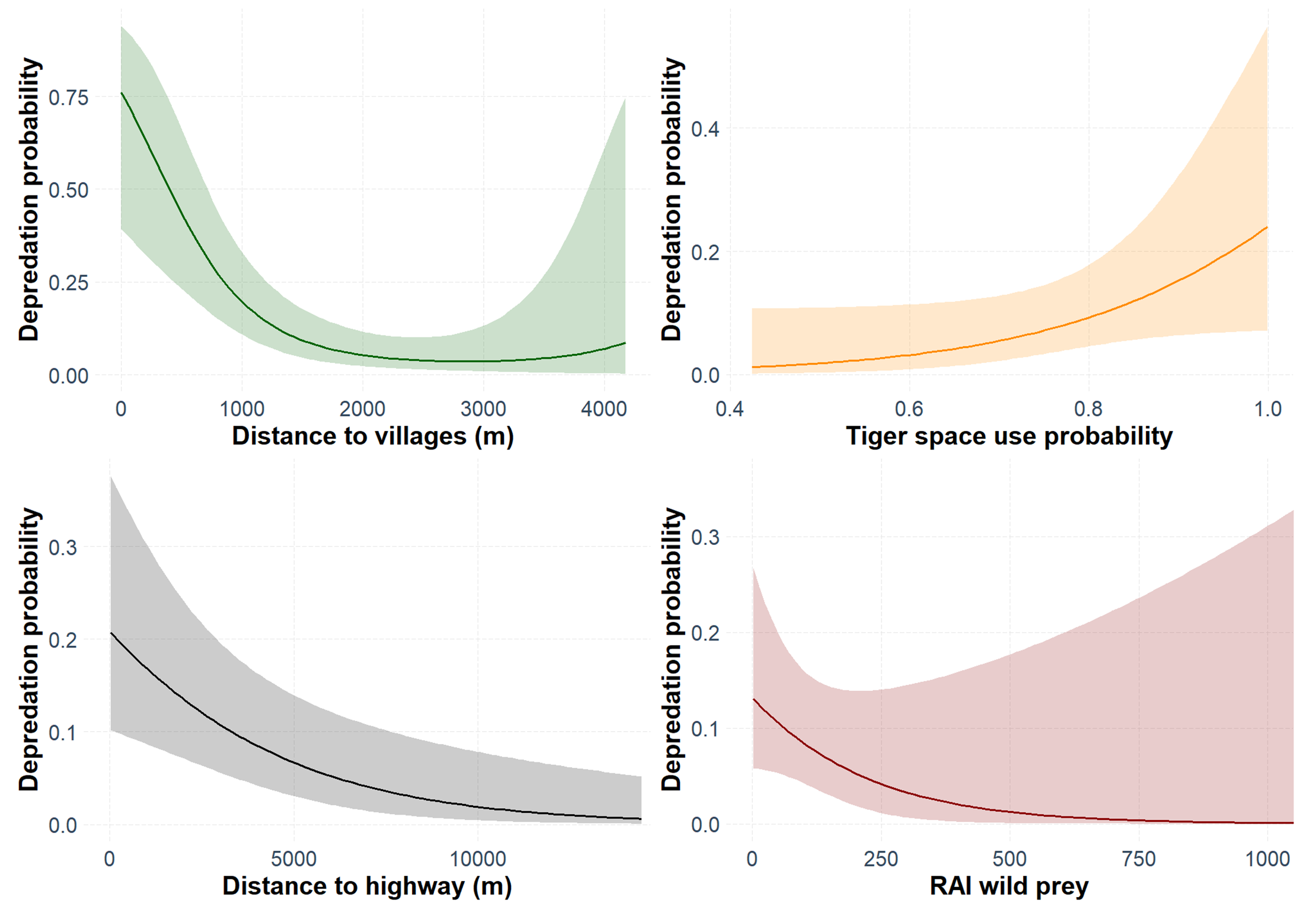

Supplement: Supplemental Information 13 — The shaded region indicates the 95% confidence interval of the response curve. [file peerj-12-17693-s013.png]

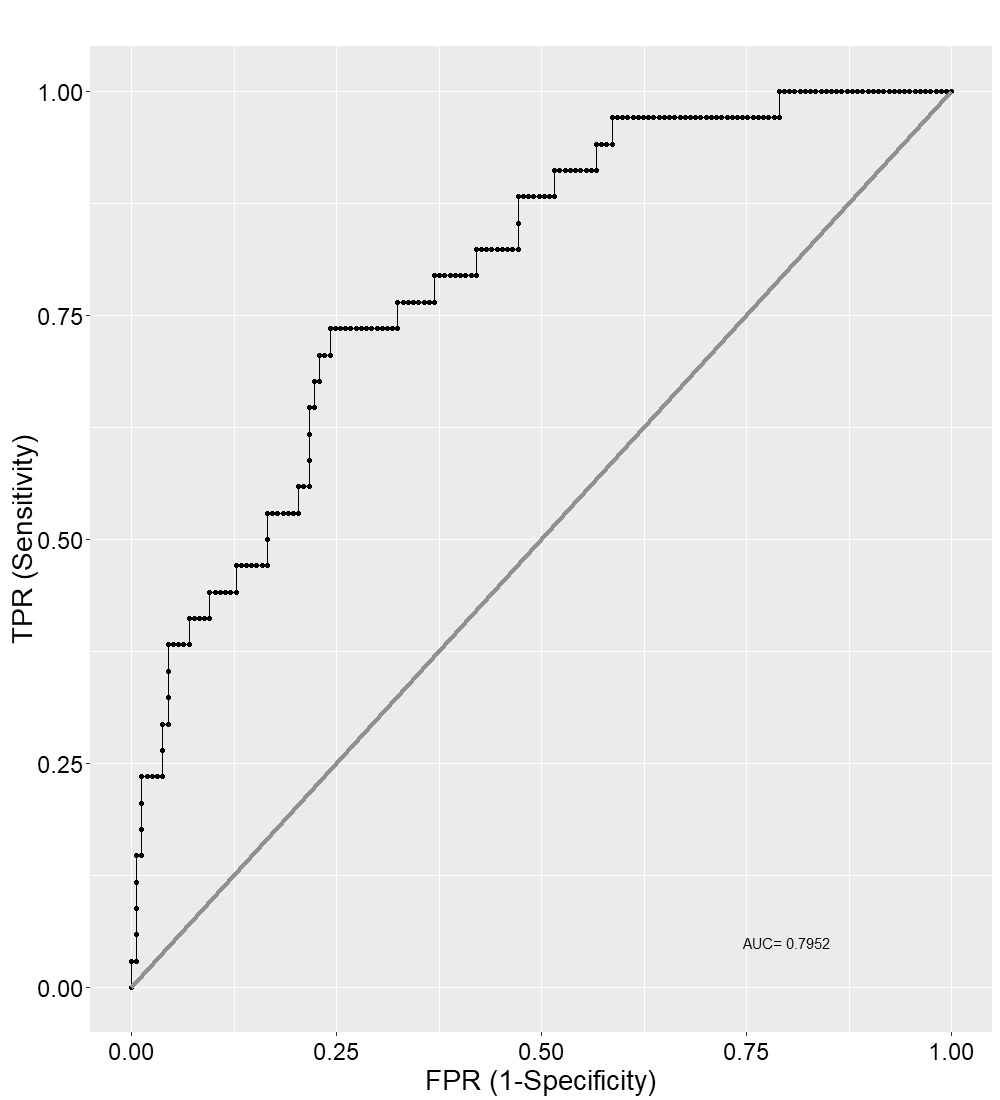

Supplement: Supplemental Information 14 — AUC = 0.7952 [file peerj-12-17693-s014.png]
